# Sequence variation and selection of small RNAs in domesticated rice

**DOI:** 10.1186/1471-2148-10-119

**Published:** 2010-04-30

**Authors:** Yu Wang, Dan Shen, Shiping Bo, Huan Chen, Jian Zheng, Qian-Hao Zhu, Daguang Cai, Chris Helliwell, Longjiang Fan

**Affiliations:** 1 Department of Agronomy, Zhejiang University, Hangzhou 310029, China; 2 CSIRO Plant Industry, Canberra, ACT 2601, Australia; 3 Department of Molekulare Phytopathologie, Christian-Albrechts-Universität zu Kiel, D-24118 Kiel, Germany

## Abstract

**Background:**

Endogenous non-coding small RNAs (21-24 nt) play an important role in post-transcriptional gene regulation in plants. Domestication selection is the most important evolutionary force in shaping crop genomes. The extent of polymorphism at small RNA loci in domesticated rice and whether small RNA loci are targets of domestication selection have not yet been determined.

**Results:**

A polymorphism survey of 94 small RNA loci (88 *MIRNAs*, four *TAS3 *loci and two miRNA-like long hairpins) was conducted in domesticated rice, generating 2 Mb of sequence data. Many mutations (substitution or insertion/deletion) were observed at small RNA loci in domesticated rice, e.g. 12 mutation sites were observed in the mature miRNA sequences of 11 *MIRNAs *(12.5% of the investigated *MIRNAs*). Several small RNA loci showed significant signals for positive selection and/or potential domestication selection.

**Conclusions:**

Sequence variation at miRNAs and other small RNAs is higher than expected in domesticated rice. Like protein-coding genes, non-coding small RNA loci could be targets of domestication selection and play an important role in rice domestication and improvement.

## Background

Endogenous non-coding small RNAs (21-24 nt) play an important role in post-transcriptional gene regulation in plants. In general, small RNAs are grouped into two major classes, microRNAs (miRNAs) and short-interfering RNAs (siRNAs) based on the mechanisms by which they are synthesized and function [1]. miRNAs are generated from a stem-loop primary transcript by the endonuclease Dicer-like 1 (DCL1) and its partners. To date, 414 miRNAs from 151 miRNA families (miRBase, http://microrna.sanger.ac.uk/sequences/, Release 14.0), including several natural antisense miRNAs (nat-miRNAs) and a mitron, have been identified in rice [2,3]. siRNAs derive from double-stranded RNA precursors that are processed mainly by the endonucleases DCL2, DCL3 and/or DCL4 [4]. Several types of siRNAs, such as trans-acting siRNAs (ta-siRNAs), natural antisense transcript-derived siRNAs (nat-siRNAs), repeat-associated siRNAs (ra-siRNAs) and miRNA-like long hairpin siRNAs, have been identified in rice and other plants [3,5-13].

mRNAs are regulated by several classes of small RNAs through base complementarity leading to cleavage or repression of translation of the mRNA. It has been observed that single nucleotide polymorphisms (SNP) within a miRNA binding site can cause significant phenotypic changes [14]. Genome-wide investigation of nucleotide variation at small RNA loci can determine the prevalence of functional variations in a species; this was first assessed in humans [15]. In plants, a large-scale survey of miRNA polymorphism has been performed in Arabidopsis [16]. This survey showed that mutations in mature miRNA sequences are rare and that the level of polymorphism in miRNAs is lower than in their flanking regions, suggesting purifying selection in the miRNA sequences. Evidence of positive selection on some miRNA loci has been found in Arabidopsis [16,17]. Our previous studies on rice miRNAs and miRNA binding sites suggested that positive selection and nucleotide mutations play an important role in co-evolution of miRNAs and their targets [18,19].

Despite the importance of small RNAs in regulation of development in crops, investigation of polymorphism of miRNAs and other small RNAs in rice and other crops is still in its infancy. The extent of sequence variation at functional regions of small RNAs and their potential contribution to phenotypic evolution during crop domestication are unclear. Unlike Arabidopsis, rice has experienced domestication and subsequent artificial genetic improvement and therefore has been shaped by an additional bottleneck effect. Rice is believed to have been domesticated approximately 10,000 years ago [20,21]. Several protein-coding genes controlling domestication traits have been identified and a notable feature of these domestication genes is that they generally encode transcription factors that regulate other protein-coding genes by direct binding to their DNA [22]. Most conserved miRNAs target transcription factors with an important role in plant development [1], but whether miRNAs and other types of non-coding small RNAs are targets of domestication selection in crops is yet to be determined.

**Figure 1:**
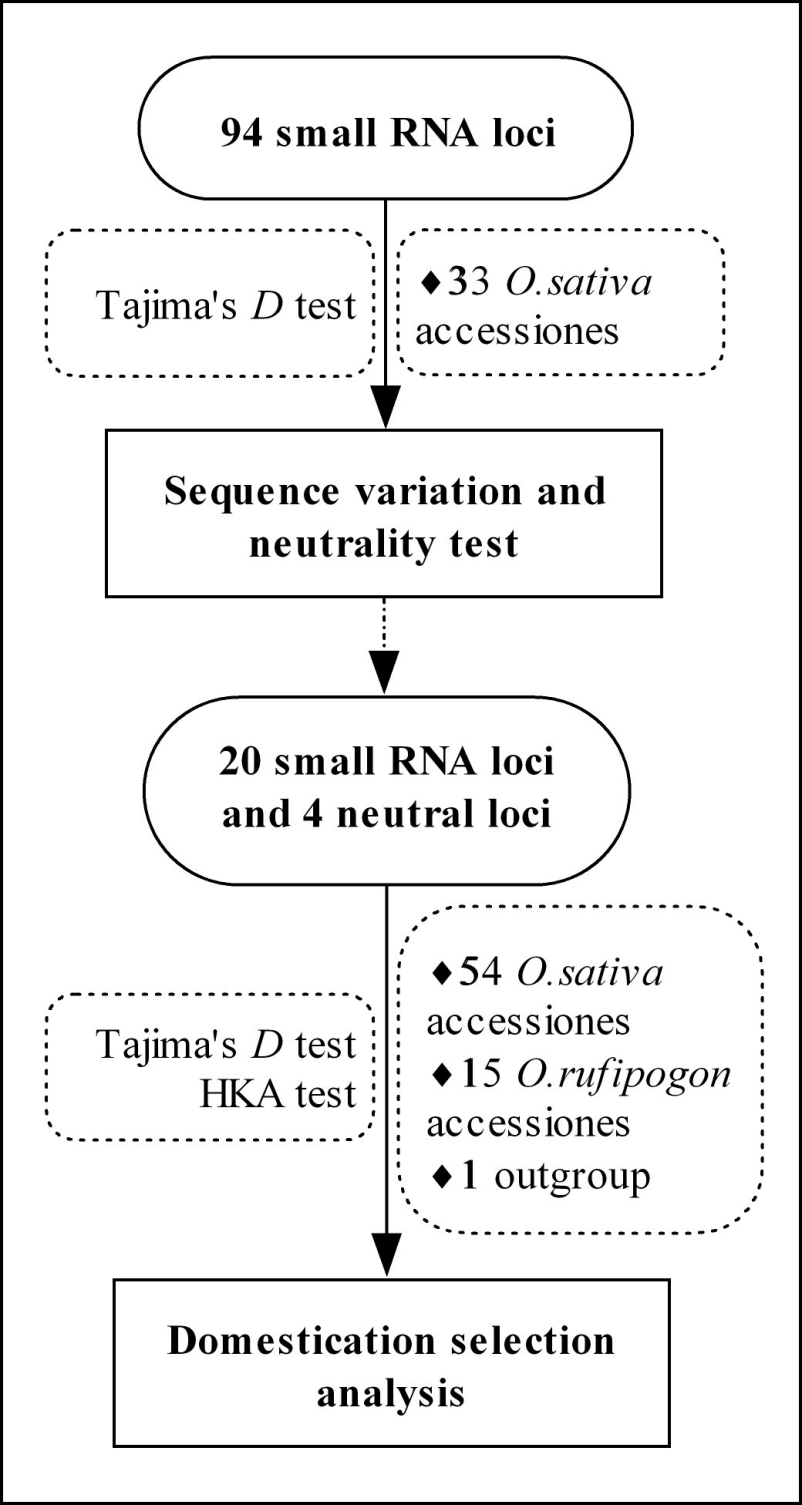
**A flow chart describing the analysis steps involved in this study**. At the first step, 94 small RNA generating loci were selected and their sequence variation and neutrality were estimated using a population of 33 domesticated rice accessions (see Figures 2, 3 and 4; Additional files 3, 4 and 5). At the second step, 20 small RNA loci were selected from the 94 small RNA loci and their sequence diversity and potential for having been under domestication selection (see Table 1) were further investigated using a cultivated population (54 accessions) and a wild population (15 accessions).

In this study, we carried out an investigation of sequence variation and selection of small RNAs in domesticated rice by two steps (Figure 1). Firstly, we selected 94 small RNA generating loci (including *MIRNAs*, *TAS3 *and miRNA-like long hairpin loci) and analyzed their sequence variation and neutrality within a domesticated rice population of 33 accessions, and secondly, we further investigated 20 of the 94 small RNA loci in populations of 54 cultivated rice accessions and 15 wild rice accessions to determine how many of these loci might have experienced domestication selection. Our results suggest a non-neutral evolutionary pattern of small RNAs in the cultivated rice and that several of them are putative targets of selection during rice domestication.

## Results

### Nucleotide divergence and neutrality of small RNAs

A total of 94 small RNA loci, including 88 *MIRNAs *from 40 miRNA families, four members of the *TAS3 *family and two miRNA-like long hairpins, were selected for a polymorphism survey of rice small RNAs (Additional file 1). With the exception of the two miRNA-like long hairpins, genomic fragments containing the intact precursor sequences of the small RNAs were amplified and sequenced from a population of 33 domesticated rice accessions (17 *indica *and 16 *japonica *cultivars), which were collected from diverse geographic locations (Additional file 2). For the two miRNA-like long hairpins which have large loop regions, the two stem regions were amplified and sequenced. Fragments from each rice accession were aligned to each other for each small RNA locus. The average fragment length of all alignments was 579.0 bp, which covered the precursor sequences of the investigated small RNAs (Additional file 3).

**Figure 2:**
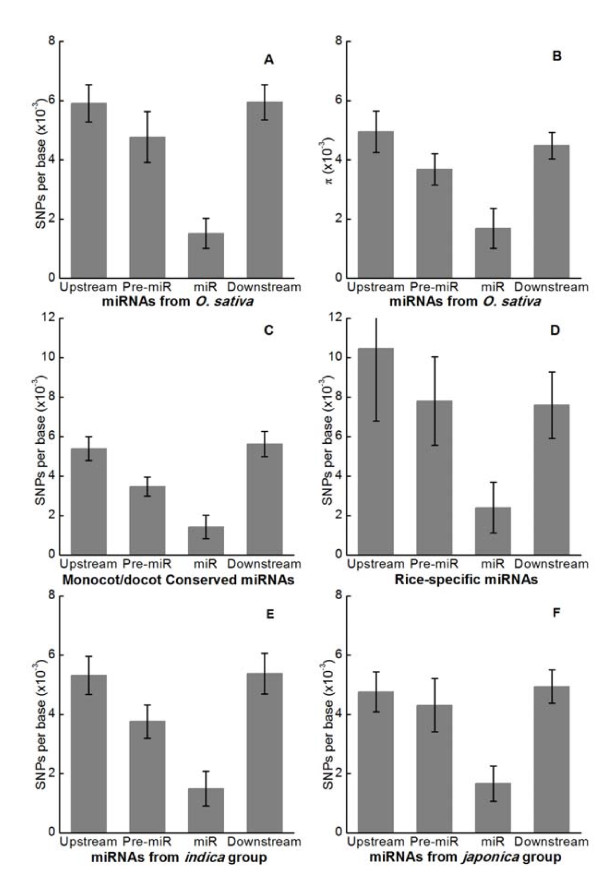
**Diversity of *MIRNAs *in cultivated rice**. **A-B**: mean numbers of SNPs per site (A) and π (B) in the mature miRNA sequences and their flanking regions. **C-D**: mean numbers of SNPs per site in the dicot/monocot conserved (C) and rice-specific (D) miRNAs and their flanking regions. **E-F**: mean numbers of SNPs per site in the mature miRNA sequences and their flanking regions in *indica *(E) and *japonica *(F) subpopulations. SE bars are shown.

**Figure 3:**
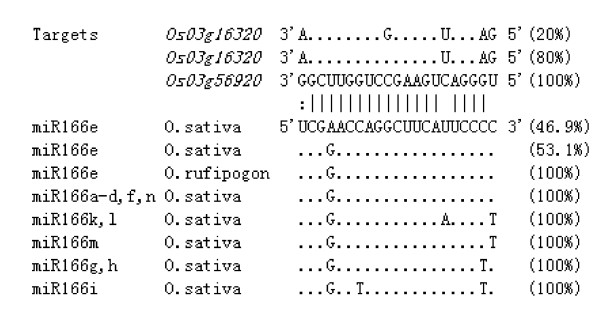
**Polymorphism of the mature sequences of the miR166 family and their targets in rice**. Base pairing between miR166e and its predicted target *Os03 g5692*0 is shown with short bars representing a Watson-Crick pair and two vertical dots representing a G-U pair. Percentages of rice accessions with different genotypes in cultivated and wild populations are shown on the right side. For further details see Additional file 3 and 4.

Of the 88 investigated *MIRNA *genes, 11 (12.5%) showed SNPs or insertion/deletions (indels) within their mature miRNA sequences in the cultivated rice population (Additional file 4), leading to an average number of SNPs per 1000 sites of 1.52 ± 0.51 (mean ± SE of the mean) or an average pairwise nucleotide diversity (π) of 0.00169 ± 0.00067 (Figure 2A and 2B). As an example, the polymorphic mature sequence of the miR166 family is presented in Figure 3 (further details are shown in Additional file 3 and 4). A SNP was observed at the fourth position from the 5' end of the mature sequences of miR166e. Out of the 32 cultivated *indica *and *japonica *accessions investigated, 17 including *indica *cultivar 93-11 and wild rice had the same miR166e sequence (with G at the fourth position) as in miRBase http://microrna.sanger.ac.uk/sequences/, while 15 had an A at the fourth position (Figure 3). In all other members of the miR166 family the fourth base is G (Figure 3). These results suggest that the fourth position A could be a recent mutation. It would be interesting to determine whether this mutation has an impact on the interaction between miR166e and its targets because it results in a perfect match with the predicted targets at the fourth position. From our sequencing data for miR166e target genes and the recently released rice SNP data http://www.oryzasnp.org[23], a SNP was found at the tenth position (counting from the 5' end of miR166e) of the miR166e binding site in *Os03 g16320*, one of the predicted miR166e targets, in 20% of accessions investigated. miRNA-mediated cleavage takes place between positions 10 and 11, a mutation from C to G at the tenth position might abolish miR166e-mediated cleavage of *Os03 g16320 *in these accessions, but whether this change has a consequence on rice depends on the specific interaction between miR166e and *Os03 g16320 *because *Os03 g16320 *could still be regulated by other members of the miR166 family.

In addition to the mature miRNA sequences, many SNPs were also observed in the flanking regions of miRNAs. Compared to the mature miRNA sequences, significantly higher SNP densities or diversities were observed in the precursor miRNA (pre-miRNA) sequences, but the highest polymorphism was found in the upstream or downstream region of pre-miRNAs (Figure 2A and 2B). These results suggest that purifying selection was the predominant evolutionary force acting on miRNA sequences in rice as reported for Arabidopsis [16]. Some miRNAs are conserved in both dicot and monocot plants while others are species-specific. Of the 88 *MIRNA *genes investigated in this study, 64 are conserved and 17 are rice-specific (Additional file 1). A dramatic difference in nucleotide diversities in the mature miRNAs and their flanking regions was observed between the conserved and rice-specific *MIRNA *loci (Figure 2C and 2D). The nucleotide diversity of the rice-specific *MIRNAs *was nearly twice that of the conserved *MIRNAs*. However, no significant difference in nucleotide diversity could be found in the miRNAs from the two subspecies of cultivated rice although the average number of SNPs per site of the *indica *subgroup was slightly lower than that of the *japonica *subgroup in both the mature miRNA and its flanking regions (Figure 2E and 2F).

Polymorphisms were also found in the functional regions of siRNA-generating loci in rice, for example, at phases P5'_5 and P5'_9 of a miRNA-like long hairpin (*AK120922*), and in ta-siARF and the 3' miR390 binding site of *TAS3a2 *(Additional file 5).

To examine neutrality of small RNAs in rice, Tajima's *D *[24], a widely used neutrality test, was employed. Tajima's *D *test measures the frequency distribution of polymorphisms and selection is expected to skew the population frequency of genetic variants relative to the neutral equilibrium model (NE). Under NE, the mean Tajima's *D *is expected to be zero and a negative value indicates an excess of rare sequence variants relative to NE expectation and a recent positive selection is thus inferred. In contrast to positive selection, balanced selection retains genetic differences and elevates the Tajima's *D *statistic towards a positive value [25].

**Figure 4:**
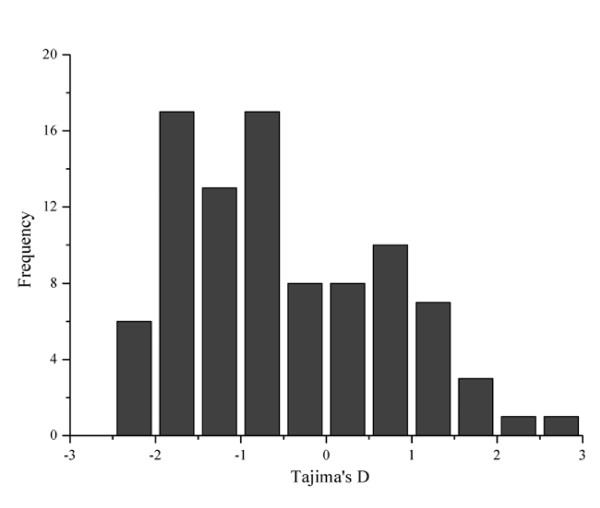
**Distribution of Tajima's *D *values of all small RNA loci in cultivated rice**.

For each small RNA locus, Tajima's *D *was calculated for the miRNA- or siRNA-containing sequence fragment. A slightly skewed distribution of Tajima's *D *values was observed in domesticated rice (Figure 4), which is similar to that observed in Arabidopsis [16]. Significant probabilities of non-neutral patterns of sequence variation were detected at several loci in one or both subgroups of the cultivated rice (Additional file 3). Significantly negative values were found for several small RNAs, such as miR395a/b and *TAS3a2 *(Tajima's *D *value: -2.21 and -2.01) in the *indica *subgroup, suggesting that these small RNA loci could have experienced positive selection during rice evolution. On the other hand, some small RNA loci possessed an extreme positive Tajima's *D *value (e.g. 1.94 for *TAS3a1*), indicating a balanced selection for these loci. The above results suggest that polymorphisms at or linked to the small RNA loci might be a result of selection.

### Potential domestication selection

*Indica *and *japonica *rice were domesticated independently and their domestication genes usually show different evolutionary histories of selection between cultivated and wild rice populations [26,27]. To find candidate small RNA loci that experienced domestication selection, 20 loci were selected from the set of 94 small RNA loci for further analysis (Figure 1) using a cultivated rice population including 29 *indica *and 25 *japonica *cultivars, a wild population including 15 accessions of *O. rufipogon *and an outgroup (Africa wild rice, *O. barthii*) (Additional file 2). Of the 20 loci selected, three (miR395a/b, *TAS3a2 *and *MIR399d*) had significant negative Tajima's *D *values and one (miR390) had extremely low divergence according to the above neutral test results (Additional file 3); the remaining loci were randomly chosen from the small RNA list shown in Additional file 1.

As previously mentioned, the extent of nucleotide diversity of selected genes tends to be reduced. Our analyses indicated that nucleotide diversity (π) of the four neutral genes (*Adh1*, *Waxy*, *ks1 *and *RGRC2*; [28,29]) reduced less than 2.6 fold in the cultivated rice population compared to their wild progenitors. Of the 20 small RNA loci, eight (*MIR164a/d/e*, *MIR166f*, *MIR390*, *MIR399d*, *MIR440 *and *AK120922*) had a nucleotide diversity reduction greater than 2.6 fold (from 2.9 to 21.2 fold) in the domesticated rice compared to their wild progenitors (Table 1). For example, nucleotide diversity of *MIR390 *reduced 7.0 and 6.3 fold in the two domesticated subgroups relative to the wild population, and nucleotide diversities of the miRNA-like long hairpin (*AK120922*) reduced 5.7 and 11.1 fold. This result suggests that these eight small RNA loci probably underwent selection during rice evolution.

**Table 1:** Sequence diversity, Tajima's *D *and HKA test results

Small RNA	*O. sativa*						*O. rufipogon*			Target	Candidate Status
						
	*Indica *subgroup			*Japonica *subgroup							
				
	*N*	*D*	HKA-*P*	*N*	*D*	HKA-*P*	*N*	*D*	HKA-*P*		
*MIR164e*	21	-1.90*	0.101	21	1.18	0.285	10	-1.04	0.447	NAC	Putative
*MIR390*	25	-1.21	0.106	20	-0.09	**0.014**	11	-1.23	0.257	*TAS3*	Putative
*MIR395a-b*	27	-1.56	NA	21	-1.94*	NA	11	-1.49	NA	APS/AST	Putative
*TAS3a2*	28	-1.86*	**0.029**	24	-0.85	0.158	10	-1.35	0.503	ARF	Putative
*AK120922*	25	0.26	**0.003**	18	-1.77	0.055	11	-2.05*	0.250	Unknown	ND
*MIR164a*	25	1.54	0.129	22	-1.31	0.215	11	-0.84	0.433	Unknown	ND
*MIR164c*	23	-0.51	0.167	23	-0.63	0.297	8	0.47	0.294	NAC	ND
*MIR164d*	25	-0.48	0.147	21	-1.29	0.260	12	-1.54	0.464	NAC	ND
*MIR166f*	29	-1.02	0.161	23	-0.81	0.342	10	-0.20	0.419	HD-ZIP	ND
*MIR395i-k*	25	0.98	0.106	18	-0.52	0.248	9	-0.60	0.245	APS/AST	ND
*MIR396f*	21	-0.54	0.089	20	0.76	0.288	10	-0.68	0.470	GRL	ND
*MIR399a*	27	0.35	0.099	24	-0.68	0.278	14	1.21	0.403	PT	ND
*MIR399d*	23	-2.37*	0.107	22	-0.87	**0.002**	12	-2.04*	0.255	PT	ND
*MIR399i*	22	-0.95	0.102	22	-1.08	0.196	11	-1.03	0.442	PT	ND
*MIR440*	25	0.04	0.097	20	-0.57	0.137	14	-1.58	0.339	Unknown	ND
*MIR443*	29	0.15	0.148	21	-1.57	0.274	9	-0.39	0.301	Unknown	ND
*MIR1318*	22	0.34	0.078	21	-0.65	0.227	12	-0.45	0.439	Unknown	ND
*MIR1432*	26	0.20	0.081	22	-1.30	0.176	10	0.57	0.382	EF	ND
*MIR1862d*	21	0.71	0.113	20	-0.14	0.270	9	-1.88*	0.394	NAC	ND
*MIR1867*	24	-1.21	0.094	24	-1.11	0.280	10	-0.94	0.493	Unknown	ND

*N*, number of accessions sequenced *D*:Tajima' *D*; HKA-*P*: *P *value of multi-locus HKA test; NA: not available; NAC, NAC domain transcription factor; *TAS3*, ta-siRNA gene using miR390 as the processing guide; APS, ATP-sulfurylase; AST, sulfate transcription factor; ARF, Auxin response factor; HD-ZIP, homeodomain leucine-zipper transcription factor; GRL, GRL transcription factor; PT: Phosphate transporter; EF: EF hand proteins; ND, not determined. The significant (*P *< 0.05) test results were labeled bold and * (for Tajima's *D *results). For details, also see Additional file 3.

To further investigate positive selection in these small RNA loci, in addition to the Tajima's *D *test, another independent test, the Hudson-Kreitman-Aguade (HKA) test [30], was performed. The HKA test is able to measure the degree of nucleotide diversity reduced by positive selection, a feature of the sequence data that cannot be measured by the Tajima's *D *test. Rejection of the multi-locus HKA test that measures the difference in diversity within species relative to divergence between species is often considered as evidence of a positive selection. The multi-locus HKA test requires an outgroup and reference loci that are believed to have not been affected by selection. For this purpose, African wild rice (*O. barthii*) that is the nearest phylogenetic node to *O. sativa *and *O. rufipogon *[31] was used as the outgroup and the aforementioned four neutral genes were used as the reference loci. Of the 20 small RNA loci, four miRNA genes (*MIR164e*, *MIR390*, *MIR395a/b *and *MIR399d*) and two siRNA genes (*TAS3a2 *and *AK120922*) had signatures of positive selection in *indica *and/or *japonica *subgroup according to Tajima's *D *or HKA test (Table 1). No positive selection was detected by the two tests for other 14 *MIRNA *loci. In the wild rice population, *MIR399d*, *MIR1862d *and the miRNA-like long hairpin (*AK120922*) presented evidence of positive selection (Tajima's *D *value of -2.04, -1.88 and -2.05, respectively), suggesting the non-neutral evolution of these three genes in the wild rice population. A gene can be considered as a candidate domestication gene if a significantly positive selection signal is detected in the cultivated population but not in the wild population [27]. Based on this criterion, our results thus suggest that *MIR164e*, *MIR390*, *MIR395a/b *and *TAS3a2 *are potential candidates of small RNA loci that have experienced direct selection during rice domestication.

## Discussion

To date, large-scale studies of nucleotide variation at miRNAs have only been carried out in human [15] and Arabidopsis [16]. Overall, our results in rice support the findings in human and Arabidopsis that purifying selection is one of the main evolutionary forces acting on rice *MIRNA *genes, maintaining lower levels of sequence divergence in mature miRNAs than in their flanking regions. In Arabidopsis, only a few substitution events were observed in the mature miRNA sequences (2 of the 66 investigated miRNAs) in a population including 24 diverse wild accessions. However, nucleotide mutations in the mature miRNA sequences were found in 11 of the 88 investigated rice miRNAs in this study. We also found polymorphisms in at least seven miRNAs in the wild rice population (data not shown). The higher percentage of SNPs or indels found in rice miRNAs implies that the polymorphism levels of miRNAs and other miRNA-like small RNAs may vary among species.

Our results suggest selection, and probably domestication selection, on some small RNA loci (Table 1 and Additional file 3). Small RNAs regulate genes that control a wide range of traits; the functions of these small RNA regulated genes might provide clues for the traits under selection via small RNA. One interesting result in this study is that both *MIR390 *and *TAS3 *are potential candidates of selection. miR390 acts as a guide for processing of *TAS3 *and biogenesis of ta-siRNAs, including the functional ta-siARF [5,10,11]. ARFs (auxin response factors) are encoded by a large gene family (25 members in rice) and are involved in regulation of a wide range of biological functions in rice [32]. ta-siARF downregulates *ARF2*, *ARF3 *and *ARF4 *[5,12] that affect gynoecium patterning [33], proper timing of vegetative shoot development and establishment of leaf polarity [34]. At least two ARFs have been detected as selected genes in maize [27,35]. It is unclear whether rice *ARF *genes have experienced selection during evolution. Our results suggest that genes involved in the same regulatory pathway could all be targeted by domestication selection for enhanced growth response and productivity. miR164e targets NAC domain transcription factors that play an important role in development, stress-tolerance and disease-resistance in rice [36,37]. miR395 targets ATP-sulfurylases that are involved in sulfate assimilation. Expression of miR395 is greatly increased under sulfate starvation conditions [38]. Sulfur is an essential macronutrient required by plants. miR395 regulated sulfate metabolism might have been selected for high sulfate usage efficiency. Our results suggest that miRNAs and other small RNA loci should not be ignored in the endeavor to identify the molecular basis of domestication and improvement of crops.

Note that there are three limitations in our effort to identify candidate small RNA loci experienced domestication selection. First, a bottleneck effect or demographic effect can also cause significant reduction of genetic diversity and it could not be excluded in our analysis. Second, the positive selection detected in this study could have arisen by selective sweep rather than direct selection on the small RNA loci themselves. Third, although we identified several mutations in the functional regions (mature miRNA, miRNA precursor or promoter) of the candidate small RNA genes in domesticated rice, such as in the mature miR395a/b sequences and in tasi-ARF and the miR390 binding sites of *TAS3 *(Additional file 4 and 5), and found distinct expression levels of miR164e in the cultivated and wild rice (data not shown), it does not necessarily indicate a direct consequence of the domestication event. Further experiments are needed to reveal the consequence of positive selection, such as phenotypic changes.

Our sequencing results revealed sequence polymorphism in miR166e and its target gene *Os03 g16320 *(Figure 3). By checking the recently released rice SNP dataset (http://www.oryzasnp.org[23]), we found a similar situation with miR443 and the target gene *Os01 g49940 *(a C → T mutation at the fifth position from the 3' end of miR443 and a G → A mutation at the eighth position from the 3' end of the miR443 binding site in *Os01 g49940*). We also observed SNPs in the miRNA binding sites of targets of miR164d/e and miR530, but no SNP was observed in the mature sequences of the two miRNAs. These results suggest that evolution of miRNAs and their targets could be independent, although a co-evolution relationship between miRNAs and their target sequences does happen in cultivated rice as demonstrated previously [18].

At least one member each of four miRNA families (miR166, miR167, miR171 and miR395) were found to have experienced positive selection based on Tajima's *D *test in the natural Arabidopsis populations [16,17]. These four miRNA families are conserved in dicot and monocot plants. In our study, significant signals of positive selection were also detected by Tajima's *D *test in at least one member of the miR166, miR167 and miR395 family in *indica *or *japonica *subgroup (Additional file 3), implying a potential conservation of adaptive evolution between rice and Arabidopsis for these miRNA families. A common feature of these three families is that they have relatively large number of family members (e.g. 23 and 6 miR395 members in rice and Arabidopsis, respectively). These homologous genes might have experienced rapid functional divergence after gene expansion. It has been suggested that positive selection acts on gene copy number variations [39]. Positive selection could promote adaptive evolution but whether plants enhance their fitness in the changing environment by adding copy number of miRNAs requires further investigation.

## Conclusions

Our survey of polymorphisms in 94 small RNA loci in rice, the first such effort in crops, revealed a higher level of sequence variations in miRNAs in domesticated rice than in Arabidopsis. In agreement with previous studies, our investigation suggests that purifying selection dominated the evolution of small RNAs in rice. A neutrality test revealed non-neutral evolution of small RNA loci in which both positive selection and balanced selection are involved. Our results suggest that domestication selection on small RNA loci could play an important role in rice domestication and/or improvement.

## Methods

### Plant Materials

Fifty-four cultivated rice accessions (*Oryza sativa*, 29 *indica *and 25 *japonica*) from a wide range of geographical locations and 15 accessions of the wild ancestor, *O. rufipogon*, were selected for investigation of nucleotide diversity in small RNA loci. Two accessions of the Africa wild rice relative, *O. barthii*, were used as an outgroup species in a neutrality test. Details of the 71 rice accessions are in Additional file 2.

### **Small RNAs selected for analyses of nucleotide diversity and domestication selection**

The majority of the *MIRNAs *selected are conserved in other species to ensure they are *bona fide MIRNAs*. For all the rice-specific *MIRNAs*, authenticity is supported by experimental evidence and BLAST analysis has been used to remove those potentially derived from repeat regions of the rice genome. The siRNAs used in this study have been experimentally identified and confirmed by our previous study [3]

### PCR and DNA Sequencing

Primers were designed based on the genomic sequence of *japonica *cultivar Nipponbare of the Rice Genome Annotation Project (http://rice.plantbiology.msu.edu/; [40,41] using the Primer3 program [42]http://fokker.wi.mit.edu/primer3/input.htm and were compared to the rice genome sequence to ensure their specificity. Primer pairs were designed to amplify products with a length of 600 - 1300 bp, including the pre-miRNA and its flanking regions (for *MIRNA *loci). Details of the primers and their products were provided in Additional file 6. Polymerase chain reaction (PCR) amplification was carried out in a DNAEngine Peltier Thermal Cycler (BIO-RAD) in a total volume of 50 μl. The reaction mixture contained 10-100 ng of template DNA, 2 μl of each primer (10-20 pmol), 0.6 μl of *Taq *DNA Polymerase (5 U/μl), 1 μl of dNTPs (10 mM), 3 μl of MgCl_2 _(25 mM), 5 μl of 10× PCR buffer (Sangon, China). PCR was performed using the following conditions: 5 min at 95°C followed by 35 cycles of 30 s at 95°C, 30 s at 54°C, and 90 s at 72°C, and a final extension at 72°C for 10 minutes. When this program failed to amplify the expected products, specific conditions were experimentally determined. For *O. sativa *cultivars, purified PCR products were directly sequenced using the forward or reverse primer. For wild rice, in which either homozygous or heterozygous individuals might exist, PCR fragments were cloned into pGEM T-Easy vector (PMD19-T, Takara) and sequenced using the forward or reverse primer. The sequencing quality files were checked manually to avoid the sequencing errors. Sequences with poor quality were re-sequenced. All sequences have been deposited into GenBank under the accession numbers GQ418390-GQ420345 and HM138917-HM140183. In total, approximately 2.0 Mb of sequences were generated in this study.

### Statistical Analysis

CLUSTALW version 1.82-UNIX was used for multiple sequence alignments [43] and segregating sites were identified manually using GeneDoc [44]. Two summary statistics parameters (θ and π) for nucleotide diversity and Tajima's *D *statistic [24] were calculated with a perl script. θ and π estimate the population mutation rate per locus based on the number of segregating sites and the mean value of pairwise divergence per locus, respectively [45,46]. Multi-locus HKA tests were performed using the HKA program available at http://lifesci.rutgers.edu/hey/home. The entire segments that contain the small RNAs were amplified by the primers shown in Additional file 6 and used for a neutrality test. For *MIRNA *loci, nucleotide diversities were separately estimated for the mature miRNAs, the pre-miRNAs, and the upstream and downstream regions of the mature miRNA (the miR395 family that is located at four clusters in which most members are less than 100 bp apart from each other was not included in this analysis). Sequences of the mature miRNAs and the pre-miRNAs were retrieved from miRBase http://microrna.sanger.ac.uk/sequences/. Conservation of miRNAs in dicots and monocots is based on Sunkar and Jagadeeswaran [47]. Targets of miRNA were predicted using Patscan [48] by searching the full length cDNA sequence dataset (release 5) of the Rice Genome Annotation http://rice.plantbiology.msu.edu with a penalty score up to four.

## Abbreviations

miRNA: microRNA; siRNA: short inferring RNA; ta-siRNA: trans-acting siRNA; *TAS3*: trans-acting siRNA locus 3; HKA: the Hudson-Kreitman-Aguade test; indel: insertion and deletion; SNP: single nucleotide polymorphism.

## Competing interests

The authors declare that they have no competing interests.

## Authors' contributions

LF designed the experiments. DS, HC and JZ performed the experiments. YW and SB analyzed the data. LF, QZ, CH, YW and DC wrote the paper. All authors read and approved the final manuscript.

## Supplementary Material

Additional file 1 **Small RNA loci investigated in this study**. 94 small RNA loci (88 *MIRNAs*, four *TAS3 *loci and two miRNA-like long hairpins) were included. Click here for file

Additional file 2 **Accession numbers and geographic origin of the cultivated and wild rice used in this study**. Fifty-four cultivated rice accessions (*Oryza sativa*, 29 *indica *and 25 *japonica*), 15 accessions of the wild ancestor, *O. rufipogon*, and two accessions of the Africa wild rice relative, *O. barthii*, were selected. Click here for file

Additional file 3 **Polymorphism of small RNAs in domesticated rice**. Number of accessions sequenced (N), length of the core alignments in which all sequences contain bases without gaps (L), total number of segregating sites (S) and two diversity parameters (θ and π) in the 94 small RNA loci of rice were shown. Click here for file

Additional file 4 **Mutations at the mature miRNA sequences in domesticated rice**. Mutations (SNP or indel) at 11 mature miRNA sequences in domesticated rice were listed. Click here for file

Additional file 5 **Mutations at the functional regions (phases) in *TAS3 *and miRNA-like long hairpin loci of domesticated rice**. Mutations (SNP or indel) at the functional regions (phases) in a *TAS3 *and a miRNA-like long hairpin loci of domesticated rice were listed. Click here for file

Additional file 6 **Primer pairs for amplification of small RNA loci**. Ninety-six primer pairs used in this study were provided. Click here for file
