# Supplementary material for: Sequence variation and selection of small RNAs in domesticated rice
Source: BMC Evol Biol. 2010 Apr 30;10:119. doi: 10.1186/1471-2148-10-119 (PMC2887405; doi:10.1186/1471-2148-10-119)
Supplement: Additional file 1 — Small RNA loci investigated in this study. 94 small RNA loci (88 MIRNAs, four TAS3 loci and two miRNA-like long hairpins) were included. [file 1471-2148-10-119-S1.DOC]

**Additional data file 1**

**Small RNA loci investigated in this study**

| Type | Family | No. of members | Conservation |
| --- | --- | --- | --- |
| miRNA |  |  |  |
|  | miR156 | 4 | Dicots/monocots |
|  | miR159 | 1 | Dicots/monocots |
|  | miR160 | 3 | Dicots/monocots |
|  | miR162 | 2 | Dicots/monocots |
|  | miR164 | 4 | Dicots/monocots |
|  | miR166 | 12 | Dicots/monocots |
|  | miR167 | 10 | Dicots/monocots |
|  | miR168 | 1 | Dicots/monocots |
|  | miR169 | 2 | Dicots/monocots |
|  | miR171 | 1 | Dicots/monocots |
|  | miR172 | 4 | Dicots/monocots |
|  | miR319 | 1 | Dicots/monocots |
|  | miR390 | 1 | Dicots/monocots |
|  | miR393 | 2 | Dicots/monocots |
|  | miR394 | 1 | Dicots/monocots |
|  | miR395 | 7 | Dicots/monocots |
|  | miR396 | 4 | Monocots |
|  | miR397 | 1 | Dicots/monocots |
|  | miR398 | 1 | Dicots/monocots |
|  | miR399 | 4 | Dicots/monocots |
|  | miR437 | 1 | Monocots |
|  | miR438 | 1 | Rice-specific |
|  | miR440 | 1 | Rice-specific |
|  | miR443 | 1 | Rice-specific |
|  | miR444 | 2 | Monocots |
|  | miR446 | 1 | Rice-specific |
|  | miR528 | 1 | Rice-specific |
|  | miR529 | 1 | Rice-specific |
|  | miR530 | 1 | Dicots/monocots |
|  | miR535 | 1 | Dicots/monocots |
|  | miR820 | 2 | Rice-specific |
|  | miR1318 | 1 | Rice-specific |
|  | miR1424 | 1 | Rice-specific |
|  | miR1430 | 1 | Rice-specific |
|  | miR1431 | 1 | Rice-specific |
|  | miR1432 | 1 | Rice-specific |
|  | miR1433 | 1 | Rice-specific |
|  | miR1439 | 1 | Rice-specific |
|  | miR1862 | 1 | Rice-specific |
|  | miR1867 | 1 | Rice-specific |
| miRNA-like siRNA | |  |  |
|  | *Os06g21900A* | 1 | Rice-specific |
|  | *AK120922** | 1 | Rice-specific |
| ta-siRNA |  |  |  |
|  | *TAS3* | 4 | Dicots/monocots |
| Total |  | 94 |  |

*Including *Os12g42380* and *Os12g42390*.
