# Supplementary material for: Sequence variation and selection of small RNAs in domesticated rice
Source: BMC Evol Biol. 2010 Apr 30;10:119. doi: 10.1186/1471-2148-10-119 (PMC2887405; doi:10.1186/1471-2148-10-119)
Supplement: Additional file 2 — Accession numbers and geographic origin of the cultivated and wild rice used in this study. Fifty-four cultivated rice accessions (Oryza sativa, 29 indica and 25 japonica), 15 accessions of the wild ancestor, O. rufipogon, and two accessions of the Africa wild rice relative, O. barthii, were selected. [file 1471-2148-10-119-S2.DOC]

**Additional data file 2**

Accession number and geographic origin of the cultivated and wild rice used in this study

| Number | ID/Accession* | Cultivar Name | Code | Variety Group# | Country |
| --- | --- | --- | --- | --- | --- |
| 1  2 | P155  P224 | Kasalath  zhenxian97 | IND1  IND2 | indica  indica | Japan  CHINA |
| 3 | P370 | Aizhizhan | IND4 | indica | CHINA |
| 4 | P84 | Guangluai4 | IND6 | indica | CHINA |
| 5 | P4 | Minghui63 | IND7 | indica | CHINA |
| 6 | P21 | Teqing | IND8 | indica | China |
| 7 | IRGC30416 | IR36 | IND9 | indica | PHILIPPINES |
| 8 | IRGC3697 | CO25 | IND12 | indica | INDIA |
| 9 | IRGC9175 | JC93 | IND13 | indica | INDIA |
| 10 | IRGC5809 | DA7 | IND15 | indica | BANKLADESH |
| 11 | IRGC7755 | KALUKANTHA | IND17 | indica | SRI LANKA |
| 12 | IRGC8231 | GIE57 | IND19 | indica | VIETNAM |
| 13 | IRGC26872 | BINULAWAN | IND22 | indica | PHILIPPINES |
| 14 | IRGC27748 | KHAO DAWK MALI 105 | IND23 | indica | THAILAND |
| 15 | IRGC43400 | ILIS AIR | IND 25 | indica | INDONESIA |
| 16 | IRGC58930 | CHHOTE DHAN | IND27 | indica | NEPAL |
| 17 | / | 93-11 | 9311 | indica | cHINA |
| 18 | IRGC 55471 | Chodongji | TEJ1 | temperate japonica | South KorEa |
| 19 | IRGC 27630 | Darmali | TEJ2 | temperate japonica | Nepal |
| 20 | IRGC 27716 | Kaw Luyoeng | TEJ3 | temperate japonica | Thailand |
| 21 | 14455 | Balila | TEJ4 | temperate japonica | ItalY |
| 22 | IRGC 40748 | Nep Hoa Vang | TEJ5 | temperate japonica | Vietnam |
| 23 | IRGC38690 | NPE 253 | TEJ6 | temperate japonica | Pakistan |
| 24 | IRGC 1107 | Ta Hung Ku | TEJ7 | temperate japonica | China |
| 25 | IRGC 33888 | Yelaik Meedon | TEJ8 | temperate japonica | Burma |
| 26 | IRGC 27869 | Chahora 144 | TRJ9 | tropical japonica | Pakistan |
| 27 | IRGC 43372 | CICIH BETON | TRJ10 | tropical japonica | Indonesia |
| 28 | IRGC 24225 | KHAO HAWM | TRJ11 | tropical japonica | Thailand |
| 29 | IRGC 2545 | KOTOBUKI MOCHI | TRJ12 | tropical japonica | Japan |
| 30 | IRGC25901 | MIRITI | TRJ13 | tropical japonica | Bangladesh |
| 31 | IRGC 6264 | N22 | TRJ14 | tropical japonica | India |
| 32 | IRGC 8261 | PADI KASALLE | TRJ15 | tropical japonica | INDONESIA |
| 33 | / | Nipponbare | Nipponbare | tropical japonica | Japan |
| 34 | IRGC1708 | FORTUNA | I5 | indica | UNITED STATES |
| 35 | IRGC 3810 | BINIRHEN | I6 | indica | PHILIPPINES |
| 36 | IRGC 8978 | BABAWEE | I7 | indica | SRI LANKA |
| 37 | IRGC 10358 | AI YEH LU | I8 | indica | CHINA |
| 38 | IRGC 26267 | TRUNG HUNG | I9 | indica | VIETNAM |
| 39 | IRGC 36019 | PADI REMBAI | I10 | indica | MALAYSIA |
| 40 | IRGC 43599 | SIGADIS | I11 | indica | INDONESIA |
| 41 | IRGC 73119 | SATHRI | I12 | indica | PAKISTAN |
| 42 | IRGC 74497 | KUNI ICHI NIAR 040172 | I13 | indica | JAPAN |
| 43 | IRGC 75323 | ANGKRONG | I14 | indica | CAMBODIA |
| 44 | IRGC 36039 | PULUT HITAM s.1072 | I15 | indica | MALAYSIA |
| 45 | IRGC 74498 | KUNIHIKARI MOCHI | I16 | indica | JAPAN |
| 46 | IRGC 2264 | SHEN BONG | J17 | japonica | KOREA |
| 47 | IRGC 9439 | SUECA | J18 | japonica | BRAZIL |
| 48 | IRGC 7410 | BUENKETAN | J19 | japonica | PHILIPPINES |
| 49 | IRGC 23747 | LAI DAWK DOO | J20 | japonica | THAILAND |
| 50 | IRGC 25725 | SIMANUK | J21 | japonica | INDONESIA |
| 51 | IRGC 34682 | BOTESHAWAR | J23 | japonica | BANGLADESH |
| 52 | IRGC 89259 | THKHU DHAN | J24 | japonica | NEPAL |
| 53 | IRGC 26908 | KARUNA | J25 | japonica | INDIA |
| 54 | IRGC 26253 | NEP BAP | J26 | japonica | VIETNAM |
| 1 | IRGC 81886 | WILD | RUF1 | *O. rufipogon* | India |
| 2 | IRGC 82988 | WILD | RUF2 | *O. rufipogon* | China |
| 3 | IRGC 83818 | WILD | RUF3 | *O. rufipogon* | Vietnam |
| 4 | IRGC 89019 | WILD | RUF5 | *O. rufipogon* | Cambodia |
| 5 | IRGC 93044 | WILD | RUF6 | *O. rufipogon* | Cambodia |
| 6 | IRGC 93208 | WILD | RUF8 | *O. rufipogon* | Nepal |
| 7 | IRGC 104819 | WILD | RUF18 | *O. rufipogon* | THAILAND |
| 8 | IRGC 103423 | WILD | RUF13 | *O. rufipogon* | Sri lanKa |
| 9 | IRGC 104308 | WILD | RUF14 | *O. rufipogon* | BURMA |
| 10 | IRGC 104624 | WILD | RUF15 | *O. rufipogon* | China |
| 11 | IRGC 106178 | WILD | RUF25 | *O. rufipogon* | Thailand |
| 12 | IRGC 104802 | WILD | RUF17 | *O. rufipogon* | Thailand |
| 13 | IRGC 105656 | WILD | RUF20 | *O. rufipogon* | India |
| 14 | IRGC 105696 | WILD | RUF21 | *O. rufipogon* | Nepal |
| 15 | IRGC 106168 | WILD | RUF24 | *O. rufipogon* | Vietnam |
| 1 | IRGC105612 | WILD | BAR1 | *O. barthii* | Zambia |
| 2 | IRGC104287 | WILD | BAR2 | *O. barthii* | Mali |

* Rice seeds were kindly provided by International Rice Research Institute (IRRI) and China Rice Research Institute. Sequences of Nipponbare and 93-11 of which genomes have been sequenced were downloaded from NCBI and TIGR, respectively.

# Variety classification of *Oryza sativa* according to Garris *et al*. (2005, Genetics)
