# Supplementary material for: Sequence variation and selection of small RNAs in domesticated rice
Source: BMC Evol Biol. 2010 Apr 30;10:119. doi: 10.1186/1471-2148-10-119 (PMC2887405; doi:10.1186/1471-2148-10-119)
Supplement: Additional file 6 — Primer pairs for amplification of small RNA loci. Ninety-six primer pairs used in this study were provided. [file 1471-2148-10-119-S6.DOC]

**Additional data file 6 Primer pairs for amplification of** small RNA loci

| No. | Small RNA | | Primer sequence | Expected length (bp) |
| --- | --- | --- | --- | --- |
| 1 | miRNA | miR156bc | F-TGGCTAGCTAATCCATGAGA  R-TCAGAAATACTTCACAGAGAGAGTACG | 891 |
| 2 |  | miR156d | F-TCACCAGCATCCGTATCACTCTCC  R-ACCCGTCCCATCCATCTCCT | 778 |
| 3 |  | miR156h | F-CTGTGCCGACATCTCTTCAA  R-TCGCTGATCTCCTCGGTAGT | 984 |
| 4 |  | miR159a | F-CGCCTAGTAGCATCTCCA  R-CAACATACATCATCACCACC | 663 |
| 5 |  | miR160a | F-AATGTATAAGTTAAGCCGTTTCAA  R-CTAGCAGCCATGGAGAGGAG | 921 |
| 6 |  | miR160b | F- GTTGGGCAGCAGCAGTAATAAGAA  R- AGAAAAAGGGGCAGCTGATAGAGA | 734 |
| 7 |  | miR160e | F- ACATCAGAAACGCAGCGAGTAAAT  R- AAAGGGAGAAAAGAAAGAGTGACA | 593 |
| 8 |  | miR162a | F-CCCACCTGTTCCAAATCCTACTCG  R-GCCAAAGCCCACAAATTACATCAA | 919 |
| 9 |  | miR162b | F-CTTACCGAAATGTCAGAGC  R-ATATTCATACGCAGCAGTTA | 796 |
| 10 |  | miR164a | F-GCCCGGCCCTTCTTCTGTCTCTT  R-CCGGCACCTCTCCTCCTCTTCC | 784 |
| 11 |  | miR164c | F- GAAATCAGCTCACGTTTCCC  R- TCGGCTAGTCAATAATCCCG | 983 |
| 12 |  | miR164d | F- CAGGCAAATTTCCACGAGTT  R- AATTGGATTGGATGGATGGA | 734 |
| 13 |  | miR164e | F- TGCTGATTGCTAATCCCTCC  R- TGTAGCGAGTTGTTGCCTTG | 990 |
| 14 |  | miR166a | F-ATGGCTCGTTTCAATAGATGC  R-TAATGAAATAGAAGAGGGGGAGAG | 749 |
| 15 |  | miR166b | F-TGCCACCAAGATCTACTCCTCCTA  R-AACTGCCCCAATTAAAAGACCAT | 702 |
| 16 |  | miR166c | F-CAACAAGGTGCCAAAAGAAG  R-GGGAACACCAATACGCTGAGT | 690 |
| 17 |  | miR166d | F-ACCCCAATCTTCTCTGTTCTC  R-ACTTTTCCCCTTTGTTTCTCCTAC | 711 |
| 18 |  | miR166e | F-CTCCCTGGACAAGCACTAGC  R-ACTCATCACCCATCAGGAGG | 992 |
| 19 |  | miR166f | 1. F-TGTGTTTTGAGAGTGGTGAATGTG   R-TTGGGTGTAGAAGTGTAGTGTGAA   1. F- TGTGTTTTGAGAGTGGTGAATGTG   R- TTGGGTGTAGAAGTGTAGTGTGAA | 670  693 |
| 20 |  | miR166h | F-GGGCGTCGATCTAAAACTACCTAA  R-ACCCGCTGTGCCAAATGGGG | 725 |
| 21 |  | miR166i | F-GCCGGCCGCCTCCTCTCTGG  R-CGCGACCTCGACCGACCTATTAT | 677 |
| 22 |  | miR166k | F-ATTGGGCGTCGATCTAAAACTACC  R-ATTGCCATTACTGCCCCTGTCCTC | 695 |
| 23 |  | miR166l | F-GGTAAGCTAGCCCCTCAATAAATG  R-GTGAGAACTGAGAAAATGAGAAGC | 748 |
| 24 |  | miR166m | F-CACCTGGGAAACGTACGAAGAAAC  R-GCTACCGCCTACCGCAAAGAAT | 618 |
| 25 |  | miR166n | F-TTTTCCGGTGTTTTGTCCTTGTAA  R-GATTTTGTGTCGCGTTCAGTTCTC | 752 |
| 26 |  | miR167a | F-AAAGCCAAGCCTGAAACACT  R-ACCCTTAATTACATACACTGAGAA | 794 |
| 27 |  | miR167b | F-TTCGATAACCCGATGAGGAG  R-ACATGGCCGCGATAAACTAC | 849 |
| 28 |  | miR167c | F-CCCACCACCACCATCACAGT  R-GAGAGGAGACGGTAGTTATTGG | 824 |
| 29 |  | miR167d | F- AGCAAGAACACCCTACAACA  R- AGCCTTCTTCTTCCTCTCAT | 766 |
| 30 |  | miR167e | F-TATGCAGTGAAAAAGGCGAGAG  R-TAGGGGGCAGTGGTTGTGAT | 811 |
| 31 |  | miR167f | F-CAAAATCCCCACTCACACCATAG  R-ACCTCTCCCAGCACAAGCAGTA | 759 |
| 32 |  | miR167g | F-AACATTAGCCTCTCCACCAAAAAG  R-TGATAGTCTGATGCGTTACCTGAT | 785 |
| 33 |  | miR167h | F-AGGCGAGATGAGATGACAAAAGAT  R-TAAGGCTGGGCAGATAACACAA | 676 |
| 34 |  | miR167i | F-GGGGTGGGTGGGCGAGAA  R-CGAGGAGGAAATGGGGGACAAA | 838 |
| 35 |  | miR167j | F-TACAGACCGGCCAGAGATAAAC  R-CAAACGCATGCAAAGACAAG | 671 |
| 36 |  | miR168b | F-ATAACAAGGCAACACATAAACA  R-GGGCCTCCATCACTCTTTAG | 790 |
| 37 |  | miR169a | F-CAAGATTCGGCACCAACCTCACTG  R-CGCTGCATATACTGTTCCCTCTGT | 771 |
| 38 |  | miR169l | F-GAGGCCCTACATAGACACAAAG  R-CGAGGATTAACCATTTAGCACA | 480 |
| 39 |  | miR171b | F- ATATCCCAACCCCCTCTTTG  R- GGAATCAAGACCACCTGCAT | 552 |
| 40 |  | miR172a | F-CTCCGGCCAATTAACACAAAGTA  R-TGGAAACGAAATGCATCTCTGAC | 863 |
| 41 |  | miR172b | F-CACCGCATCAATCCTACT  R-CTACCTCGCTGTTGTCTC | 1108 |
| 42 |  | miR172c | F-TCGAATCTCCACGATGAACA  R-CGGGCCCTATAAATTCTGCT | 717 |
| 43 |  | miR172d | F-CTAGCTCCCACCATGACTTTATTA  R-TGGCGTATGTGGTTCTTTTCTTGT | 717 |
| 44 |  | miR319b | F-AACGAGGTGTTTGCAGTGTG  R-CCTCTTGCACCCATCTTTGT | 759 |
| 45 |  | miR390 | F-ACACCCATCACTCGTAGC  R-TTCATCCACCTCTTGTCG | 911 |
| 46 |  | miR393b | F-ATTCGTTCTCAAAGTAAAAG  R-GCCAGGGGTAGAGTCGTA | 993 |
| 47 |  | miR394 | F-CGGCTACTCCCAATACCAAA  R- GCACAAAACCCAAACCCTAA | 970 |
| 48 |  | miR395ab | F-ACGTGGCTCTCAGTCTTGTG  R-CACAGTGCTCTGCTGGTGAT | 884 |
| 49 |  | miR395i-k | F-AAAGGTCTTGGTTGGTTGTTAC  R-TCTGCGCTGTGAGGCTGAA | 1097 |
| 50 |  | miR395t | F-TGGTTCAAACACCGGTTACA  R-TGGTCCACATCAAAAACGAG | 1034 |
| 51 |  | miR395v | F-CATGCTCATTTGACCTGCAT  R-AGAACGCACGTTCATGTCAC | 967 |
| 52 |  | miR396b | F- CCCTTGGATTGATGAG  R- GTGGGAAACAGTATGG | 1095 |
| 53 |  | miR396c | F-TTTACGGAAGAGACATCAA  R-TGCTGCAACCTCATTTAGA | 831 |
| 54 |  | miR396d | F-CTTCACTCCTCCGTCCTCTG  R- GAGAGCCAAAAACCATCCAA | 932 |
| 55 |  | miR396f | F- TGTGAGAGACCTACCGGGTC  R- TTGATCAGCTTCCTCTGGCT | 850 |
| 56 |  | miR397b | F-AGAGCGCCTTAATTTCAGTTTTGT  R-AGCGTATGCATGATTCTTTTGTTC | 853 |
| 57 |  | miR398a | F-GGGGGTCACCGAAATCC  R-GAACACAGCCTTAATAGTCTT | 868 |
| 58 |  | miR399a | F-AGCCGAGTCGAATACCTAAGATGC  R-CCAACGCCTGAAACAAGTAAAAGA | 797 |
| 59 |  | miR399i | F- TACAGCTTCAAGAGCACCCA  R- CTTGGTCTGACAACCCCAGT | 949 |
| 60 |  | miR399j | F-GCTGTGCAAAGTGCCAACAG  R- CGCAACCCACAGGGTGCCAT | 1052 |
| 61 |  | miR399d | F-GAAAGGCACAAGAGGCACACTACT  R-CGCCCAGACTTCGTTTACTTTTC | 781 |
| 62 |  | miR437 | F-GAGGGCGGGGCTGTTGGTT  R-CGTTTGCGTCCTGATTTTCCTGA | 749 |
| 63 |  | miR438 | F-GCGCCACTGTGCTAGCCTCCTTC  R-CTTCCTAATGCCACCTCTCACAAT | 893 |
| 64 |  | miR440 | F-TAGCTTCGTCACTCTTATCCA  R-TGCACGCCATGTTCCTATTT | 911 |
| 65 |  | miR443 | F-ATCTCCATTCGCCTTTATCTATCA  R-CCTTTTGGGTTTATGGCTTTTC | 829 |
| 66 |  | miR444a | F-ATCCGATATGACACGCTAA  R-AATCCCTATGGAAGAGCC | 1150 |
| 67 |  | miR444d | F- TACTGGAGCACTAGACAAAAAC  R- TGAGACAGGCCAAACTTAT | | 704 |  | | --- | --- | |  | | |
| 68 |  | miR446 | F-TCCCCATGCGCATACGGGTAAT  R-ACACAGCATGCACTGAAACTGG | 865 |
| 69 |  | miR528 | F-TGGCGCACACGACACGA  R-TGAATTTTACAACGGCATACG | 712 |
| 70 |  | miR529b | F-TTTTGGGATGGAGGGTG  R-ATCGCATGAGTTTAGGG | 990 |
| 71 |  | miR530 | F-AGCTGCATGCTCATTGATTG  R- GGTCTTGGACGAGAAAGCAG | 728 |
| 72 |  | miR535 | F-TCATGCGAAAAGGCTCACTA  R-TACGGGTTTTCTTGGTTGCTATTA | 739 |
| 73 |  | miR820a | F-CGTTCCTTACCGATCTTGCT/  R-GTTCAACCCTGATGGCACTT | 985 |
| 74 |  | miR820b | F-CACTCATCGTCGTGTCCG  R-GAAATCGTTACCCATCTTA | 1009 |
| 75 |  | miR1318 | F- GAAGCAGCGCTCGGGTTGGT  R- AATGGCCGCCCCTTCCTCCT | 890 |
| 76 |  | miR1424 | F-TAATGGTAGCCTCGCATTCC  R- ACGGTGTACGGCGAAATTAG | 767 |
| 77 |  | miR1430 | F-TTGGCTTAAAATGGCGTGTCA  R-ATAAAGGCGTCGGCAAATCTAAAT | 821 |
| 78 |  | miR1431 | F-ACATGGCAACCGTCAAT  R-TACCTCTCCCAAGTGCTAATG | 603 |
| 79 |  | miR1432 | F-GAGCGCCATGTTCTTGAG  R-AAATGGCCGCCCCTTCCT | 757 |
| 80 |  | miR1433 | F-GATCGCCACCTTTTGCTTGTC  R-TACTCCGGTGCTGCTCTGTTTTAT | 704 |
| 81 |  | miR1439 | F-TTTGGTCCCGTAGCTCAGTT  R-GAAATGGGGCATCAATGAAA | 811 |
| 82 |  | miR1862d | F-AGGGGGCAGAATATACAGAC  R- TGCGCAATGCAGGCTTCGAG | 995 |
| 83 |  | miR1867 | F-TGGTCAGGCGGTGTTAGCAC  R-CGCTGACATGCTCACGGTGG | 1097 |
| 84 | ta-siRNA | *TAS3-a1* | F-TGCAGGTTCGTGACTGAAAG  R-TGTTCCCAACAGTGAGCAAG | 877 |
| 85 |  | *TAS3-a2* | F- CAAAGTTCCAAACCCTCTTGTC  R-CAGCACCATTTGGTGAGTGA | 875 |
| 86 |  | *TAS3-b1* | F- CCACCCTTCTTCACCACACT  R- CTCACAGTCTCACGGACGAA | 996 |
| 87 |  | *TAS3-b2* | F- ATGGCCTTCTTCTCCACAGA  R- ATCGTCCTACATCCGTCCTG | 831 |
| 88 | miRNA-like siRNA | *Os06g21900* 5' arm | F-GTAAATGTACTTTCCAGCAT  R-AGAAATACCTCGCCATAC | 650 |
| 89 |  | *AK120922* 5' arm (*Os12g42380*)  3’ arm  (*Os12g42390*) | F-AGGACTGGTGACAAGGCAAG  R-CATATCCACCGGACTCAAGG  F-AGGCAAGAAAGGGGAAGAAG  R-ATTGTTTGGCGACCTCTACG | 709  738 |
| 90 | Reference gene | *Waxy* | F-ACGACGCAACCACGGTAA  R-GGGCTGGAGAAATCAACAAG | 852 |
| 91 |  | *Adh1* | F-TCCCGTGTTCCCTCGGATCTTC  R-GTCACACCCTCTCCAACACTCT | 853 |
| 92 |  | *Ks1* | F-TTTCCTGGTATGCTTAGCCTTGC  R-GCGAAAAGCCATTGCACATGTTG | 941 |
| 93 |  | *RGGC2* | F-TGTTGCACTGATGGAGGCTA  R-TTTCATGACGGTCTTCTCCTG | 1000 |
